# The remote allosteric control of Orai channel gating

**DOI:** 10.1371/journal.pbio.3000413

**Published:** 2019-08-30

**Authors:** Yandong Zhou, Robert M. Nwokonko, James H. Baraniak, Mohamed Trebak, Kenneth P. K. Lee, Donald L. Gill

**Affiliations:** Department of Cellular and Molecular Physiology, The Pennsylvania State University College of Medicine, Hershey, Pennsylvania, United States of America

## Abstract

Calcium signals drive an endless array of cellular responses including secretion, contraction, transcription, cell division, and growth. The ubiquitously expressed Orai family of plasma membrane (PM) ion channels mediate Ca^2+^ entry signals triggered by the Ca^2+^ sensor Stromal Interaction Molecule (STIM) proteins of the endoplasmic reticulum (ER). The 2 proteins interact within curiously obscure ER-PM junctions, driving an allosteric gating mechanism for the Orai channel. Although key to Ca^2+^ signal generation, molecular understanding of this activation process remain obscure. Crystallographic structural analyses reveal much about the exquisite hexameric core structure of Orai channels. But how STIM proteins bind to the channel periphery and remotely control opening of the central pore, has eluded such analysis. Recent studies apply both crystallography and single-particle cryogenic electron microscopy (cryo-EM) analyses to probe the structure of Orai mutants that mimic activation by STIM. The results provide new understanding on the open state of the channel and how STIM proteins may exert remote allosteric control of channel gating.

## Abstract 2

Plasma membrane Orai channels are activated by endoplasmic reticulum calcium-sensing STIM proteins through a dynamic coupling mechanism within specialized inter-membrane junctions. This Primer explores new structural studies that hint at the extraordinary complexity of this crucial association that is critical to the generation of calcium signals.

The ubiquitously expressed Orai family of highly Ca^2+^-selective channels mediate Ca^2+^ entry signals crucial in controlling transcription and growth in all cell types [1]. The plasma membrane Orai channels are activated through a dynamic intermembrane interaction with the endoplasmic reticulum (ER) Stromal Interaction Molecule (STIM) proteins [2], which function as sensors of ER luminal Ca^2+^ [3]. The question of how these 2 proteins couple within ER- plasma membrane (PM) junctions is critical to understanding how Orai channels generate Ca^2+^ signals and their relationship to disease states [1,4,5].

The dimeric STIM proteins unfold when ER luminal Ca^2+^ decreases, becoming trapped in ER-PM junctions where they tether and activate hexameric Orai channels in the junction [3] (Fig 1A). A small (100 amino acid) tightly folded helical STIM-Orai Activating Region (SOAR) in the STIM1 dimer binds to Orai1 and is sufficient to cross-link and activate Orai1 channels [3,6,7]. Orai channels have 4 transmembrane (TM) helices, of which the C-terminal innermost TM1 is the pore-forming helix, and the outermost TM4 extension (TM4_e_) provides a strong binding site for SOAR (Fig 1B) [1]. Activation was thought to involve a 2-pronged interaction in which strong SOAR binding to the Orai1 TM4_e_ helix facilitated a further weak SOAR interaction with the N-terminal extension of the pore-forming TM1 helix that could pull the channel open (Fig 1C; left) [7,8]. More recent work suggests SOAR binding to the Orai1 C terminus alone “remotely” induces channel activation by allosteric propagation of the TM4_e_ binding signal across the channel, resulting in opening of the Orai1 TM1 pore helix to allow Ca^2+^ flow and cross-linking of channels (Fig 1C) [9, 10]. The nature of this allosteric control of Orai1 channels is the subject of some interesting recent structural studies [11–13].

**Fig 1:**
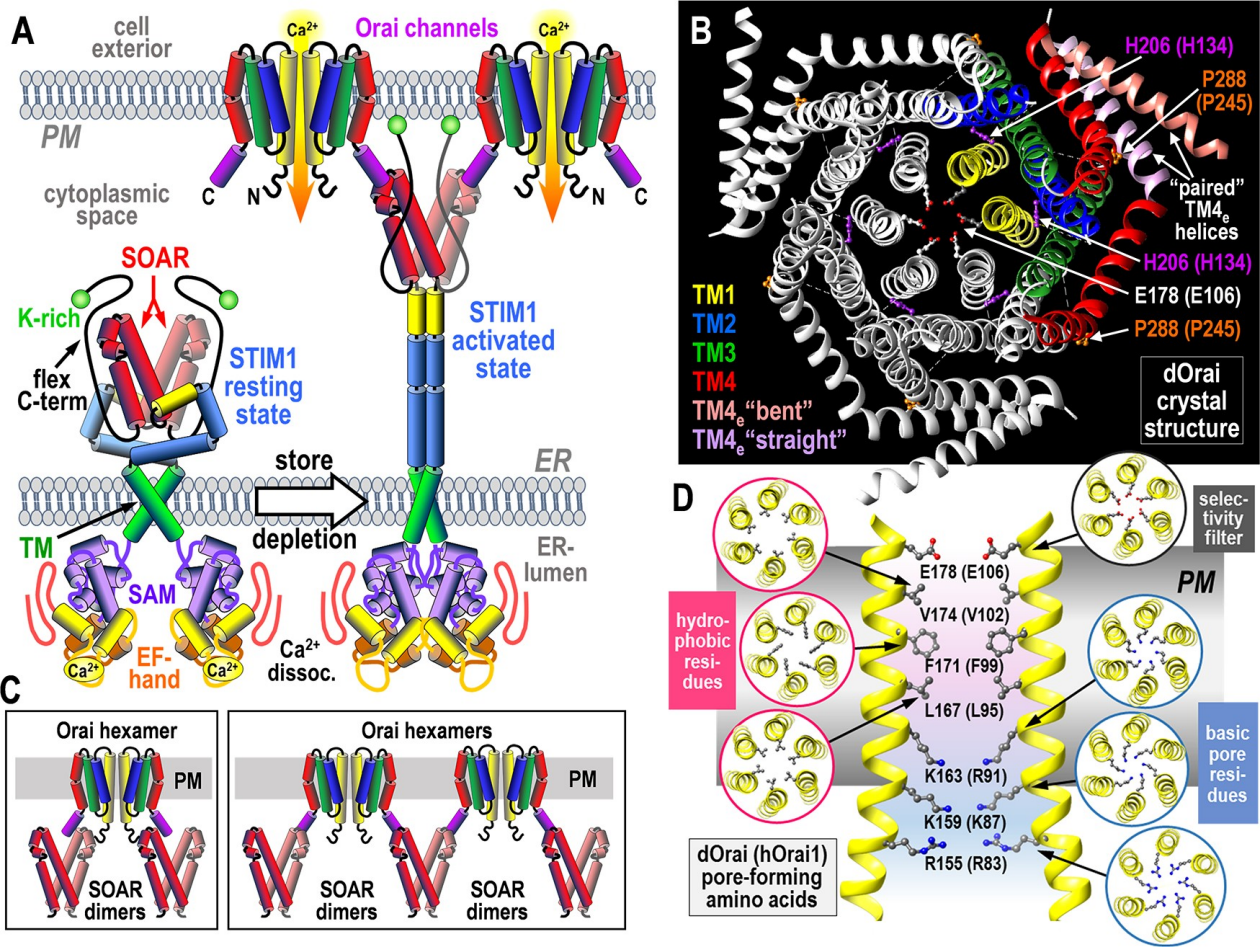
Molecular basis of STIM-induced Orai channel activation. (A) STIM proteins in the ER membrane are activated by luminal Ca^2+^ depletion and dissociation of Ca^2+^ from their N-terminal Ca^2+^-sensing EF-hand/SAM domains. Unfolding of the cytoplasmic domain allows the lysine-rich (K-rich) flexible C terminus attach to the PM to trap STIM proteins in ER-PM junctions. Exposed SOAR tether and activate Orai channels, mediating Ca^2+^ entry signals crucial in controlling gene expression, growth, secretory, and motile responses. (B) Crystal-derived structure of the dOrai channel comprising a hexameric arrangement of Orai channel subunits, each with 4 TM helices. One dOrai subunit dimer is highlighted in color. The TM1 central pore includes the outer Ca^2+^ selectivity filter, E178 (E106). The TM2 and TM3 helices assemble closely around the pore. The peripheral TM4 helix includes an outer TM4 extension (TM4_e_) that may pair asymmetrically with an adjacent subunit TM4_e_, each pair comprising an antiparallel association between TM4_e_ helices in a “bent” and “straight” configuration. dOrai mutations at 2 critical residues, H206 and P288, give rise to constitutively active channels. Residue numbers are for dOrai (hOrai1 in parentheses). (C, left) Model of the gating interaction between the active dimeric SOAR unit in STIM and the hexameric Orai channel (only 2 subunits shown) in which each SOAR unit interacts across the N-terminal TM1 and C-terminal TM4_e_ of Orai to directly gate the pore. (C, right) Alternative model in which SOAR binds only to the TM4_e_ helix and remotely induces pore gating through an allosteric conformational change propagated across the TM helices; each SOAR dimer can activate and cross-link 2 Orai subunits in separate channels. (D) The TM1 pore-lining residues in dOrai (hOrai1) that form the selectivity filter and hydrophobic and basic regions of the pore. dOrai, *Drosophila* Orai; ER, endoplasmic reticulum; hOrai, human Orai; PM, plasma membrane; SAM, sterile-alpha motif; SOAR, STIM-Orai Activating Region; STIM, Stromal Interaction Molecule; TM, transmembrane; TM4_e_, TM4 extension.

The crystallographic structure of the *Drosophila* Orai (dOrai) channel (closely related to human Orai1), in its closed state, was earlier solved by Long and colleagues (Fig 1B and 1D) [14,15]. The hunt to understand the structural basis of STIM-induced Orai channel opening has resulted in 2 recent papers from the laboratories of Long [11] and Shen [12] showing open dOrai structures. Both studies are based on mutations in the dOrai channel resulting in constitutive channel opening that may mimic the action of STIM binding. Although interesting, we must ask to what extent these structures relate to the actual physiological gating of the Orai channel by STIM proteins.

The E106 residue in the pore-forming TM1 helix of Orai1 functions as the outer selectivity filter (Fig 1D). Immediately beneath this filter, the pore is lined by 3 rings of hydrophobic resides (V102, F99, L95), the first 2 of these forming a powerful hydrophobic gate [13]. Elegant mutational studies and molecular dynamics simulations indicate that the precise positioning of these residues, particularly the large F99 headgroup, is critical to STIM1-induced channel gating [16]. A powerful Orai1 gain-of-function mutation (H134A) in the closely juxtaposed TM2 helix [17] reveals a critical TM2-TM1 coupling either by hydrogen bonding [17] or more likely a steric brake mechanism [18]. The H134A mutation induces rotation of the TM1 pore helix and displacement of the F99 residue within the pore to allow Ca^2+^ permeation through the channel [13]. Channel-activating H134 mutations with small side chains mimic STIM1 gating and are not further activated by STIM1, but they differ in not showing fast Ca^2+^-dependent inactivation which depends on STIM1. Bulkier nonactivating H134 mutations block the action of STIM1. This TM2-TM1 coupling may represent the final allosteric step triggered by STIM1. But what is the initial step? And what is the nature of the remote control process activated by STIM1 at the Orai1 TM4_e_ and allosterically propagated across the Orai1 protein to open the TM1 pore?

In recent crystallization studies, Long and colleagues examined the H206A mutation in the dOrai TM2 (equivalent to H134A in hOrai1) to try to detect the structural basis of its channel activation [11]. We might have expected rather subtle changes in the pore structure, perhaps in the hydrophobic gate. Unexpectedly, the crystallographic structure of dOrai-H206A reveals a drastically dilated lower region of the TM1 pore (Fig 2A–2C). This region is lined with 3 juxtaposed rings of basic residues (Fig 1D), the lowest of which, R155, is splayed out by about 10 Å in the dOrai-H206A structure (Fig 2C and 2E). The hydrophobic gating region might also be slightly dilated (Fig 2C and 2E), but the lower resolution of the crystal structure (6.7 Å) did not provide clear information on sidechain conformation.

**Fig 2:**
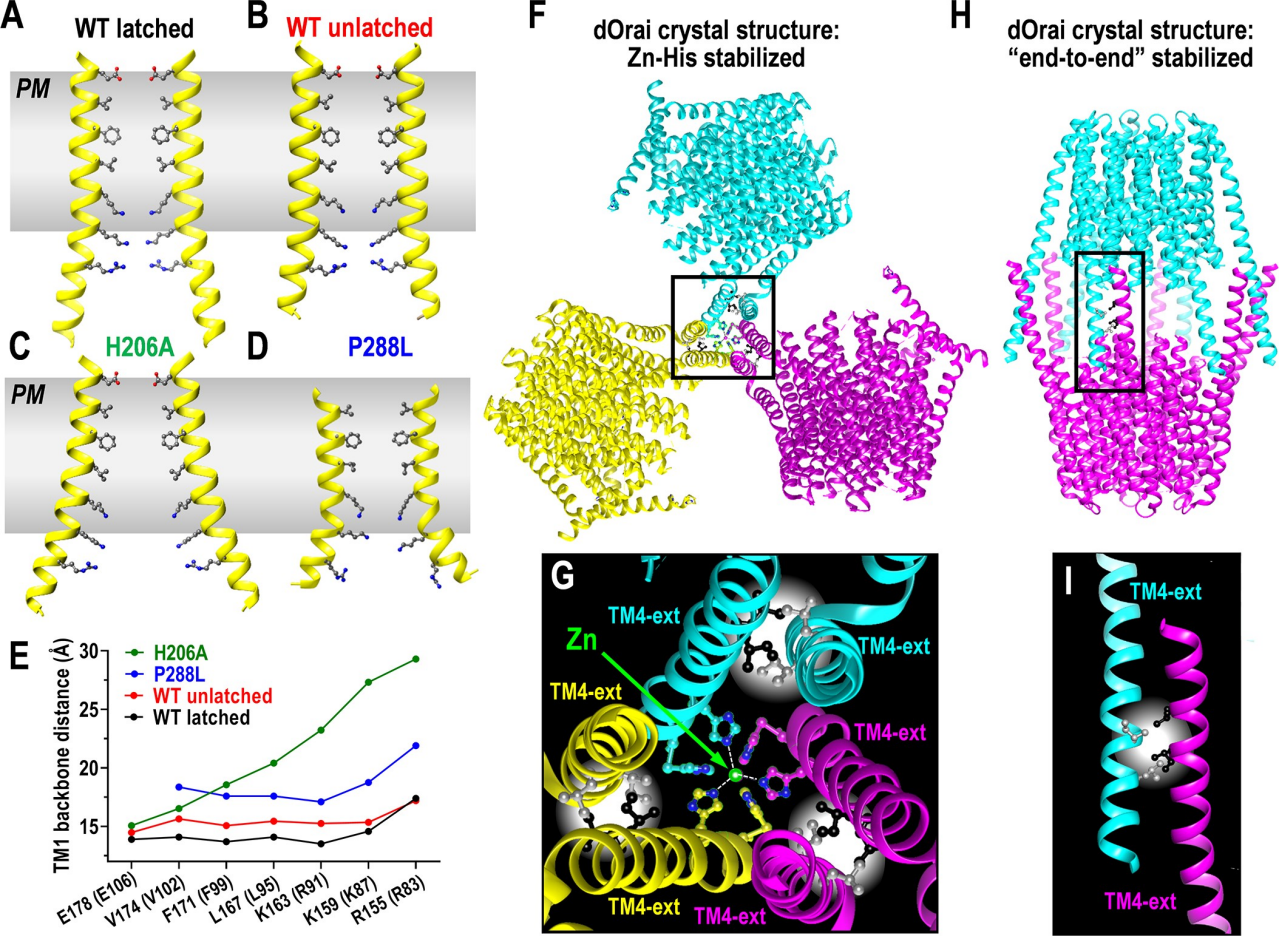
**(A–D) Crystal-derived structures of the TM1 pore-forming helix in dOrai.** Structures are shown for the WT dOrai channel derived from (A) the latched/paired dOrai WT closed-channel crystal structure [14]; (B) the unlatched/unpaired dOrai WT closed-channel crystal structure [11]; (C) the unlatched/unpaired dOrai-H206A open-channel crystal structure [11]; (D) the unlatched/unpaired dOrai-P288L open-channel crystal structure [11]. (E) Distances between the peptide backbones of the pore-lining residues across the opposite-facing TM1 helices for each of the pore structures shown in (A–D). (F) Crystal packing of the dOrai WT channel in a latched/paired configuration stabilized through Zn^2+^-mediated trimeric association of His-pairs at the C terminus of TM4_e_ helices [14]. (G) Detailed structure of the Zn^2+^-mediated trimeric association of TM4_e_ helices in (F) showing the Zn^2+^-binding to H330 and H334, and the antiparallel association of I316 and L319 residues in the crystal structure. Note the 3 His-pairs are from only 3 of the 6 TM4_e_ helices, and their Zn^2+^-binding may contribute to the asymmetric pairing of TM4_e_ helices observed in the crystal structure. (H) Crystal packing of the dOrai WT channel in a unlatched/unpaired configuration stabilized through “end-to-end” TM4_e_ interactions [11], similar to the crystal packing identified for dOrai-P288L [12]. (I) Detailed structure of the association of TM4_e_ helices in the “end-to-end” crystal packing in (H) showing the antiparallel association of I316 and L319 residues. dOrai, *Drosophila* Orai; PM, plasma membrane; TM, transmembrane; TM4_e_, TM4 extesion; WT, wild type.

In the original closed wild-type dOrai1 crystal structure, the TM4_e_ helices of adjacent subunits were captured in an antiparallel “paired” arrangement (Fig 1B) [14]. In contrast, the mutated dOrai-H206A structure revealed an extraordinary unpairing of the TM4_e_ helices and an “unlatching” (partial dissociation) of the TM4-TM3 interaction allowing the TM4 helices to separate away from the rest of the channel (see Fig 3A and 3B) [11]. The authors suggested that this unpairing/unlatching was necessary for the channel to open—thus, the paired/latched TM4 structure of the closed channel was suggested to sterically prevent dilation of the TM1 in the open, mutant pore. But, quite enigmatically, the authors also determined unpaired/unlatched crystal structures for both the closed dOrai-WT channel and also the constitutively closed dOrai-K163W mutant [11]. Thus, unlatching was certainly not sufficient for pore opening. As discussed below, it remains unclear whether the observed unlatching in these crystal structures corresponds to physiological STIM-induced channel activation.

**Fig 3:**
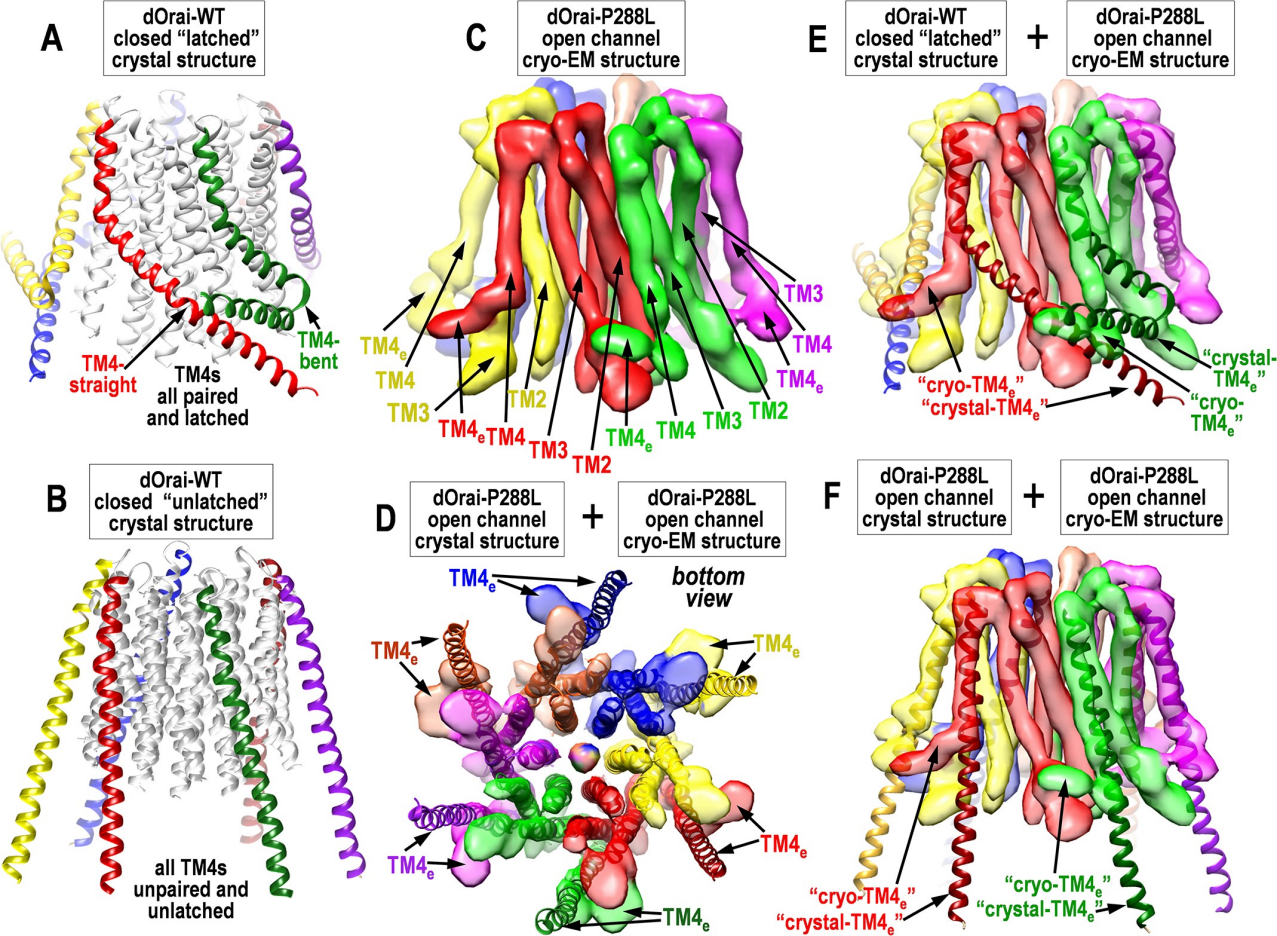
Details and comparison of crystal- and cryo-EM-derived dOrai structures. (A) Side view of dOrai WT crystal structure of closed, “latched” channel, from the Zn^2+^-stabilized, paired-TM4_e_ configuration [14]. TM4s (colored) have 3-fold symmetry because of antiparallel pairing of adjacent “straight” and “bent” TM4_e_ configurations. (B) Side view of dOrai WT crystal structure of closed, “unlatched” channel from the “end-to-end”-stabilized configuration [11]. TM4s (colored) have 6-fold symmetry, are unpaired, straightened, and mostly detached from the channel core. (C) Side view of the cryo-EM structure of the dOrai-P288L open channel [12]. Note TM4_e_ helices appear unpaired, bent in a clockwise direction, and closely associated with TM3s in adjacent subunits. Adjacent TM4s appear nonidentical, giving some outer 3-fold symmetry. (D) Comparison of bottom (cytosolic) view of crystal- and cryo-EM–derived structures of the dOrai-P288L open channel [12]. The crystal structure is from the end-to-end, unlatched configuration. Note there is close structural agreement for the inner core TM1, TM2, and TM3 helices, but unlatched TM4 helices are detached for the crystal structure and differ greatly, especially at the C-terminal TM4_e_ helices that are critical STIM-binding sites. (E) Comparison of side views of the crystal-derived latched, closed channel (from panel A, but only TM4s shown) [14] and the cryo-EM–derived dOrai-P288L open channel (from panel C) [12]. Note the remarkable apparent difference in lower TM4 configuration—the straight anticlockwise-oriented TM4_e_ in the crystal structure is bent and oriented clockwise in the cryo-EM structure. The bent TM4_e_ in the crystal structure remains so in the cryo-EM structure but appears displaced clockwise. Both TM4_e_s appear closely associated with TM3s in adjacent channel subunits. (F) Comparison of side views of the crystal-derived and cryo-EM-derived structures for the dOrai-P288L open channel (as in panel D) [12]. Only TM4s are shown for the crystal structure. Note the upper TM4s appear to be in close agreement, but the lower TM4s (including TM4_e_s) are strikingly different—straight and unconnected for the crystal structure, and bent and associated with adjacent TM3s in the cryo-EM structure. cryo-EM, cryogenic electron microscopy; dOrai, *Drosophila* Orai; STIM, Stromal Interaction Molecule; TM, transmembrane; TM4_e_, TM4 extension; WT, wild type.

A new crystallization study from Shen’s laboratory [12] approached understanding of the dOrai open channel using a different mutational model. This study utilized a mutation of the P288 residue in the TM4 helix of dOrai. This proline forms a kink in the TM4 helix in close proximity to the TM4_e_ STIM-binding site (see Fig 1B). Earlier studies identified the equivalent proline in human Orai1 (P245) as a critical residue, mutation of which leads to channel gain-of-function associated with tubular myopathy and a Stormorken-like syndrome [19]. The P245L mutation was shown to induce a constitutively active open state for the Orai1 channel with properties similar to that induced by STIM1 [20]. Likely, straightening of the TM4 helix induces a configurational change that at least partially mimics the binding of STIM1. The equivalent P288L mutation in dOrai induced constitutive channel opening closely resembling STIM-mediated activation [12]. Purification and successful crystallization of the dOrai-P288L mutant revealed a structure with some surprising similarity to the H206A structure. The dOrai-P288L had similarly straightened, unpaired TM4/TM4_e_ helices, which in this case might be expected from the proline substitution. Interestingly, Shen’s dOrai-P288L structure also reveals a prominently dilated pore, and like the pore dilation observed in Long’s dOrai-H206A crystal structure, this was mostly within the basic residue-lined, lower region (Fig 2D and 2E). However, in Shen’s case, this dilation was smaller and largely restricted to the lowest basic residue, R155 (compare Fig 2C and 2D). Again, because the resolution was not sufficient to show sidechains, it was not possible to determine whether there were additional subtle changes in the upper hydrophobic core or selectivity filter.

How this positively charged pore region contributes to Ca^2+^ ion permeation is enigmatic. It was suggested that anions in the channel may shield the positive charges to allow Ca^2+^ permeation [11,14,15]; however, mutations to neutralize the positive charges block rather than enhance Ca^2+^ channel activity [9,12,21]. Shen argues that dilation of the basic pore region draws anions into the pore not only to neutralize basic residues but also to bind with Ca^2+^ ions and assist their passage through the pore [12]. This anion recruitment model provides a new perspective on the apparently enigmatic role of the basic residues in the pore.

Whereas the recent crystal structures are giving consistent new information on the inner core structure of dOrai (TM1, TM2, and TM3), it seems that data on the periphery of the channel, that is, the STIM-interacting helix (TM4), are uncertain and may reflect changes because of crystallization conditions. Of particular interest is the question of whether TM4_e_ helices are truly paired in native resting Orai channels. The crystal lattice in the initial dOrai structure was stabilized through a Zn^2+^-mediated trimeric association of His-pairs located at the far C terminus of each TM4_e_ (Fig 2F and 2G) [14]. The antiparallel pairing of the TM4_e_ appears to involve hydrophobic interactions between the I316 and L319 residues in dOrai (equivalent to L273, L276 in hOrai1). But could this interaction be induced by crystal packing forces? Could the Ile/Leu pairs merely be proximal as a result of a Zn^2+^-induced pairing? If unpairing of TM4_e_ helices was important for STIM interaction, it might be expected that mutating the residues to prevent pairing would facilitate STIM binding. In fact, modifying either of these residues completely blocks both STIM1-binding and Orai1 channel activation [22]. Moreover, it is clear that cross-linking the TM4_e_ helices prevents Orai channel activation by STIM [23].

In contrast to the former Zn^2+^-stabilized, paired TM4_e_ structure [14], the recent crystal structures from both Long and Shen show completely unpaired/unlatched and elongated TM4 helices with unpaired TM4_e_ helices (Fig 2H) [11,12]. Unexpectedly, this is seen for both the wild-type closed channel and for each of the 2 mutated open channels (H206A, and P288L) [11,12]. We wonder whether these new “end-to-end” dimeric dOrai crystal structures shown by both Long and Shen are reasonable facsimiles of functional Orai channels. Certainly, such C-terminally interacting structures could not occur in vivo. The crystal structures are stabilized by pairing of the 12 TM4_e_ helices in the dimeric structure—6 from each Orai hexamer (Fig 2H and 2I). These interhexamer interactions involve antiparallel binding of the same I316 and L319 residues (Fig 2I) involved in intrahexamer pairing of TM4_e_ helices in the latched structure (Fig 2G). This end-to-end interaction is strong enough to straighten the TM4-P288 residues in wild-type dOrai. In addition, it is strong enough to “unlatch” another potentially important interaction—that between TM4 and TM3. Indeed, Long concludes that unlatching the TM4-TM3 interaction is necessary for channel activation. However, this contrasts with 3 recent reports showing that the TM4-TM3 interaction is necessary for channel activation [9, 12, 24], and that blocking the TM3-TM4 interaction inhibits whereas cross-linking TM4-TM3 enhances channel activation by STIM [9]. We would suggest that forced TM4-TM3 unlatching in the end-to-end crystal structures is unlikely to be part of the gating mechanism. However, the exact nature of the STIM1-induced conformational change in TM4 awaits structural proof.

The profound pore structural change observed with the H206A mutation in TM2 is remarkable (Fig 2C). This does not appear to be a direct consequence of unlatching because the unlatched wild-type dOrai channel shows little change in pore structure (Fig 2A and 2B) [11]. Yet, we have to ask whether the rather unnatural, unlatched dOrai configuration (Fig 3B) might influence the degree of pore dilation observed in response to the H206A or P288L mutations. It is possible that an important but barely detectable movement in the hydrophobic gate is magnified in the pore basic region when the channel is unlatched. New light on the uncertainty from the crystal-structure studies comes from the first published single-particle analysis of the dOrai channel by cryo-EM [12]. Even though at relatively low resolution (5.7 Å), the new cryo-EM analysis of the constitutively active dOrai-P288L channel structure reveals some remarkable similarities and differences from the crystal structure (Fig 3C and 3D). The TM1, TM2, and TM3 core helices are highly consistent with the crystal structure (Fig 3D), whereas the outer TM4 helices appear profoundly different. In contrast to the crystal data, the cryo-EM structure of dOrai-P288L reveals no evidence of TM4 straightening or TM4_e_ pairing (Fig 3C–3F), although there is some indication of 3-fold symmetry and nonidentity of adjacent TM4_e_ helices. The TM4s do not appear to be dissociated from TM3s (Fig 3C) and seem quite close to the cytoplasmic face of the membrane. Quite enigmatically, although the TM4s remain attached in the open P288L cryo-EM structure, they appear to be in a different orientation (Fig 3C, 3E and 3F). Thus, in the closed crystal structure, the TM4s are closely associated with TM3s in the same dOrai1 subunit. But, in the cryo-EM structure of the P288L open channel, 3 of the TM4s appear to be displaced clockwise and interact with the adjacent subunit (Fig 3E and 3F). If true, this would be a major divergence from each of the crystal structures; however, Shen and colleagues provided no cryo-EM structure for the closed channel; thus there is no means to assess how channel opening altered the structure. Also, the lack of clear resolution of the TM4_e_ helices in the open cryo-EM structure means we cannot yet draw firm conclusions on their configuration.

This is the “first taste” of cryo-EM structural analysis for the dOrai channel, and although difficult to draw firm conclusions, raises some intriguing questions on channel opening. The crystal- and cryo structures agree well for the channel pore and inner core (TM1, TM2, and TM3; see Fig 3D), but there is much uncertainty on the critical external STIM-binding helix formed by TM4 that allosterically triggers channel activation. The unpairing and unlatching of TM4s in dOrai remains of uncertain relevance to channel opening and is not supported by the initial cryo-EM structure. Long and colleagues suggest that pairing of TM4s and latching with TM3 keeps the channel closed and that unlatching and release of TM4_e_ helices is necessary to allow channel opening. But identification of the unlatched configuration with a closed channel (Fig 3B) militates against this conclusion, and we suggest the “massive” unlatching seen in the crystal structures may not be physiological. Indeed, the cryo-EM structure (Fig 3C) indicates a much more latched open-channel configuration than the crystal structure.

Overall, the allosteric Orai channel activation by STIM proteins remains a structural enigma—a surprising fact considering how simple and local the STIM-Orai interactions appears to be [9,25] Thus, it seems just one-half of the small dimeric SOAR region of STIM1 interacts with the short C-terminal TM4_e_ of Orai1. Of course, this interaction occurs remotely from the pore itself, and the intramolecular allosteric propagation of the signal is difficult to discern. Recent studies indicate that the Orai1 cytoplasmic loop-2 and the N-terminal TM1 domains play critical roles in propagating the STIM1-induced signal [24,26]. Crucial to understanding gating will be to obtain Orai structures with the critical channel-activating SOAR units attached. Careful comparison of high-resolution cryo-EM structures for closed and SOAR-bound open Orai channels may reveal how the channel is held in a stable closed configuration and how STIM1 association with TM4_e_ triggers the allosteric conformational switch that is conveyed across the TM3 and TM2 helices to induce the opened-pore configuration of TM1.

## Abbreviations

dOrai: *Drosophila* Orai
cryo-EM: cryogenic electron microscopy
ER: endoplasmic reticulum
hOrai: human Orai
PM: plasma membrane
SAM: sterile-alpha motif
SOAR: STIM-Orai Activating Region
STIM: Stromal Interaction Molecule
TM: transmembrane
TM4_e_: TM4 extension
